# Nanogel tectonic porous 3D scaffold for direct reprogramming fibroblasts into osteoblasts and bone regeneration

**DOI:** 10.1038/s41598-018-33892-z

**Published:** 2018-10-25

**Authors:** Yoshiki Sato, Kenta Yamamoto, Satoshi Horiguchi, Yoshiro Tahara, Kei Nakai, Shin-ichiro Kotani, Fumishige Oseko, Giuseppe Pezzotti, Toshiro Yamamoto, Tsunao Kishida, Narisato Kanamura, Kazunari Akiyoshi, Osam Mazda

**Affiliations:** 1 Department of Immunology, Kyoto Prefectural University of Medicine, Kyoto, Japan; 2 Department of Dental Medicine, Kyoto Prefectural University of Medicine, Kyoto, Japan; 3 Department of Polymer Chemistry, Graduate school of engineering, Kyoto University, Kyoto, Japan; 4 Ceramic Physics Laboratory, Kyoto Institute of Technology, Kyoto, Japan

## Abstract

Transplantation of engineered three-dimensional (3D) bone tissue may provide therapeutic benefits to patients with various bone diseases. To achieve this goal, appropriate 3D scaffolds and cells are required. In the present study, we devised a novel nanogel tectonic material for artificial 3D scaffold, namely the nanogel-cross-linked porous (NanoCliP)-freeze-dried (FD) gel, and estimated its potential as a 3D scaffold for bone tissue engineering. As the osteoblasts, directly converted osteoblasts (dOBs) were used, because a large number of highly functional osteoblasts could be induced from fibroblasts that can be collected from patients with a minimally invasive procedure. The NanoCliP-FD gel was highly porous, and fibronectin coating of the gel allowed efficient adhesion of the dOBs, so that the cells occupied the almost entire surface of the walls of the pores after culturing for 7 days. The dOBs massively produced calcified bone matrix, and the culture could be continued for at least 28 days. The NanoCliP-FD gel with dOBs remarkably promoted bone regeneration *in vivo* after having been grafted to bone defect lesions that were artificially created in mice. The present findings suggest that the combination of the NanoCliP-FD gel and dOBs may provide a feasible therapeutic modality for bone diseases.

## Introduction

A large number of elderlies suffer from bone diseases that are associated with bone resorption and low ability of bone remodeling. Non-union of the fractured bone after osteoporotic fracture at a spine or a femoral neck may cause locomotive disability or continuing bedridden state. Surgical removal of a bone tumor may seriously reduce the quality of life (QOL) and activity of daily living (ADL) of the patients. Alveolar bone resorption due to periodontal disease may cause loss of teeth, potentially resulting in systemic infection, eating disturbance, malnutrition, and dementia^1,2^. Autologous bone graft has been performed to treat a patient with severe bone loss, but the preparation of a sufficient amount of bone for transplantation may cause adverse events such as pain in the patients at the sacrificed donor site^3^. In this context, an efficient regenerative therapy to treat bone loss and bone resorption disorders is strongly needed.

To realize an effective regenerative therapy for bone diseases, it is important to build a three-dimensional bone tissue in culture by combining two elements: (i) a 3D scaffold that could be built in a tailor-made manner to have the specific size and shape of the bone loss lesion in each patient, and (ii) a considerable number of autologous osteoblasts with a high bone forming ability. The 3D scaffold should also have appropriate chemical and physical features so that the osteoblasts efficiently settle onto the scaffold and produce bone tissue. Recently, functional hydrogels have been developed and the hydrogel scaffolds induced cellular adhesion and/or function^4^.

The nanogel is nanometer-sized hydrogel which consists of a naturally occurring polysaccharide, pullulan, modified with a cholesterol moiety^5^. The nanogel is biodegradable, and safely introduced into human. The nanogel has been reported to provide a feasible drug delivery system that enables efficient transfer of short interfering RNA into tumor cells^6^, slow-release of a cytokine *in vivo*^7^, etc. Through the nanogel tectonics, we devised chemical cross-linking of the nanogel and succeeded in generating the nanogel-cross-linked (NanoClik) gel with various size, shapes and chemical characteristics^8,9^. Furthermore, freeze-thawing of the NanoClik gel resulted in nanogel-cross-linked porous (NanoCliP) gel with highly porous structure^8^. Thus, further modification of the NanoCliP gel may provide a quite suitable scaffold for osteoblasts.

Direct reprogramming technologies enable phenotypic conversion of a somatic cell type into another without passing through an intermediate pluripotent state^10–13^. Recently we succeeded in reprogramming human fibroblasts into osteoblasts (directly converted osteoblasts; dOBs) by transducing four genes each encoding Runx2 (R), Osterix (X), Oct3/4 (O), and L-Myc (L), or three genes (X, O and L)^14–16^. As fibroblasts can be easily obtained from a small piece of biopsy sample of a patient without any invasive procedure^17^ and expanded into an enough large number^18,19^, our procedure may be quite adequate for preparation of a large number of autologous osteoblasts with a high bone forming ability.

Therefore, we tried to combine the above two technologies, the nanogel tectonic material and dOBs, to generate a feasible tissue engineering procedure for a novel regeneration therapy of bone diseases. In the present study, we newly developed NanoCliP-FD gel from the NanoCliP gel that we previously reported^8^, and examined its potential as a scaffold for bone tissue engineering using the dOBs induced by our previously reported procedure^14–16^.

## Results

### dOBs adhered to and proliferated onto the fibronectin-coated NanoCliP-FD gel

**Figure 1:**
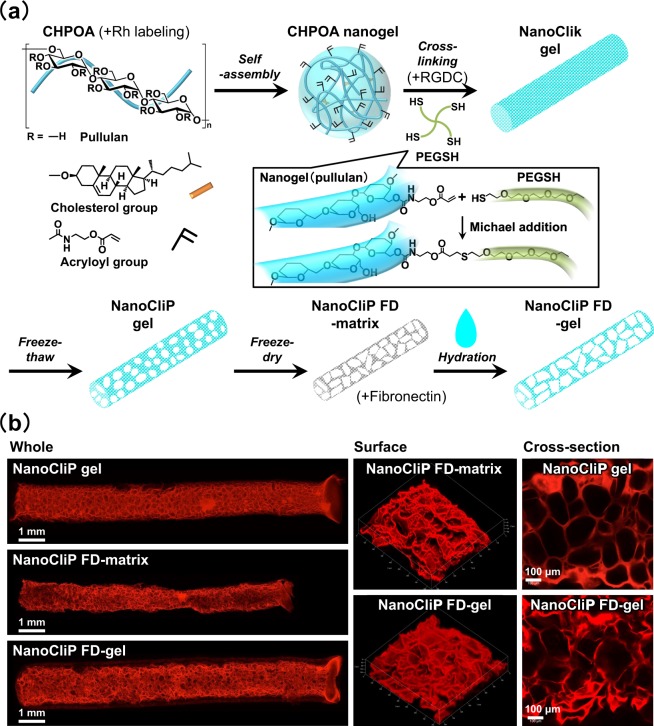
The NanoCliP-FD gel used in the study. (**a**) Preparation of the NanoCliP-FD gel. Rhodamine-labeled or non-labeled CHPOA was used to generate rhodamine-labeled and non-labeled NanoCliP-FD gel. (**b**) Rhodamine-labeled NanoCliP gel, NanoCliP-FD matrix, and NanoCliP-FD gel were prepared as described in (**a**). The 3D-composited images of the whole and surface of the samples as well as cross-sectional images of the samples were obtained by CLSM.

We modified the NanoCliP gel that we previously reported^8^ by first freezing and drying it, and subsequently by hydrating it. By doing so, we obtained a novel nanogel tectonic material, namely, the NanoCliP-FD gel (Fig. 1a). CLSM imaging of rhodamine-labeled NanoCliP and NanoCliP-FD gel revealed that the former had interconnected pores of several hundred micrometers in diameter, while the pores in the latter irregularly interlinked into larger pores (Fig. 1b and Supporting Information Table S1).

**Figure 2:**
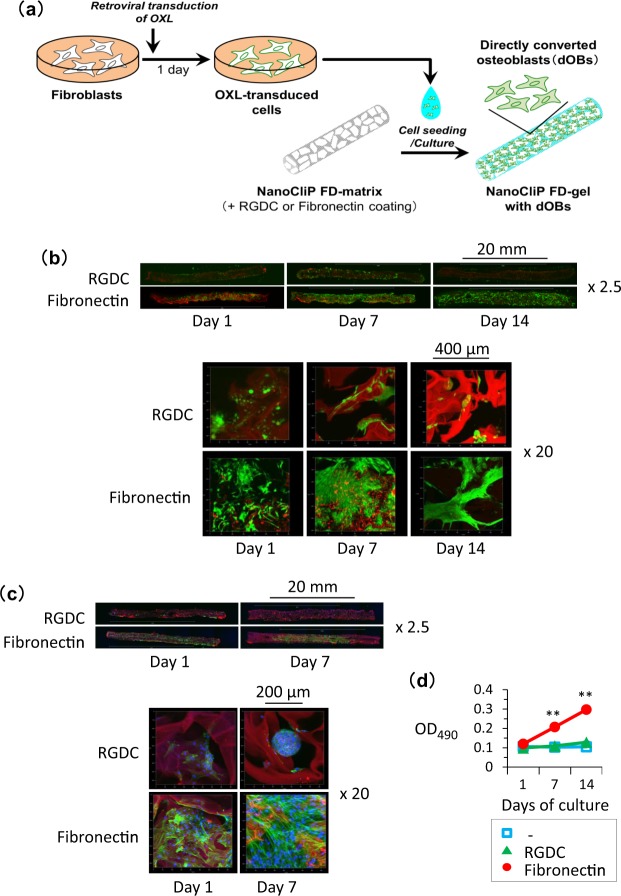
Osteoblasts efficiently adhered to and proliferated on the fibronectin-coated NanoCliP-FD gel. (**a**) Preparation of NanoCliP-FD gel with dOBs. (**b**,**c**) XOL-transduced cells were seeded into rhodamine-labeled NanoCliP-FD gel that had been coated with either RGDC or fibronectin. After culturing for the indicated days, the samples were stained with calcein-AM (**b**) or phalloidin/Hoechst 33342 (**c**), and CLSM images at magnifications of objective lenses of x2.5 (upper) and x20 (lower) are shown. (**d**) XOL-transduced cells were seeded into non-coated (−), RGDC-conjugated, and fibronectin-coated NanoCliP-FD gel. After culturing for the indicated days, cell viability was evaluated by tetrazolium-based assay. Values are means ± SD. N = 3. **p < 0.01 vs. day 1.

The rhodamine-labeled NanoCliP-FD gel was coated with either RGDC peptide or fibronectin, and the XOL-transduced cells were seeded into the gel (Fig. 2a). After culturing for 1~7 days, the distribution of the cells was observed (Fig. 2b). On day after cell seeding, viable cells positively stained with calcein-AM were widely dispersed all over the fibronectin-coated NanoCliP-FD gel. The density of the viable cells increased with elapsing culture period; and, 7 to 14 days after the initiation of the culture, the cells almost covered the entire surface of the walls of the pores in the fibronectin-coated NanoCliP-FD gel, so that the red signal of rhodamine was masked. Consistently, visualization of F-actin using fluorescent phalloidin also demonstrated that a large number of cells broadly distributed in and adhered to the walls of the pores in the fibronectin-coated NanoCliP-FD gel at day 1, and almost the entire surface was occupied by cells at day 7 (Fig. 2c). In contrast, calcein-AM-positive viable cells did not adhere to the surface in the RGDC-conjugated NanoCliP-FD gel at day 1. The cells were rarely observed in the NanoCliP-FD gel, and the red signal of rhodamine remained clearly visible, even at days 7 and 14 (Fig. 2b). Fluorescent phalloidin staining showed sphere-like aggregation of the cells, suggesting that the cells adhered to each other rather than to the RGDC-conjugated scaffold (Fig. 2c).

Cell growth was examined by a tetrazolium-based assay. As shown in Fig. 2d, the XOL-transduced cells significantly proliferated in the fibronectin-coated NanoCliP-FD gel but not in the RGDC-conjugated NanoCliP-FD gel.

These results suggest that the fibronectin coating enabled the XOL-transduced cells to adhere to and proliferate in the NanoCliP-FD gel.

### Bone matrix production by dOBs in the fibronectin-coated NanoCliP-FD gel in culture

**Figure 3:**
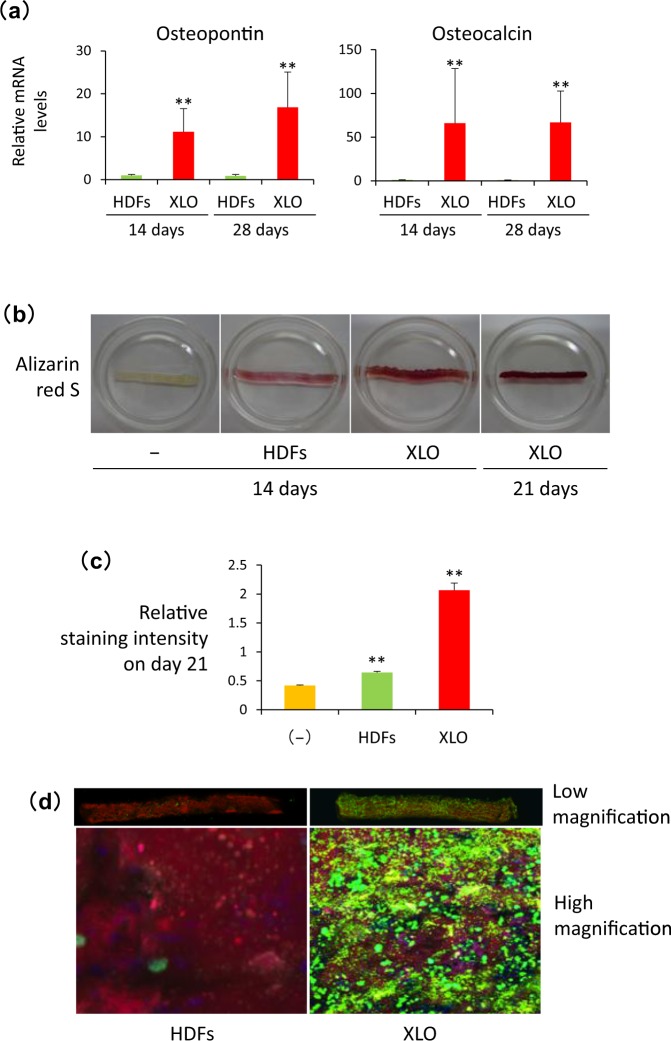
XOL-transduced cells were successfully converted into dOBs that produced calcified bone matrix in fibronectin-coated NanoCliP-FD gel. (**a**) RNA was extracted from the HDFs or XOL-transduced cells cultured in fibronectin-coated NanoCliP-FD gel for 14 and 28 days. mRNA levels for the indicated genes were evaluated by real time-RT-PCR. Values are means ± SD. N = 3. **p < 0.01 vs. HDFs. (**b**,**c**) Fibronectin-coated NanoCliP-FD gel with HDFs and XOL-transduced cells were cultured for the indicated days and stained with Alizarin red S. Some aliquots of the fibronectin-coated NanoCliP-FD gel were cultured without cell seeding (−). Macroscopic images (**b**) and relative staining intensities (**c**) are shown. Values are means ± SD. N = 3. **p < 0.01 vs. cell-free control. (**d**) Fibronectin-coated NanoCliP-FD gel with HDFs or XOL-transduced cells was cultured as above, and osteoimage assay was performed 21 days later. Confocal LSM images at low (upper) and high (lower) magnifications are shown.

We examined whether the XOL-transduced cells gave rise to dOBs during the culture in the fibronectin-coated NanoCliP-FD gel. Quantitative RT-PCR analysis was performed to evaluate expression levels of mRNA for osteopontin and osteocalcin that are osteoblast-specific proteins composing the bone matrix. As shown in Fig. 3a, mRNA for these genes was highly expressed in the XOL-transduced cells, but not by HDFs, in the fibronectin-coated NanoCliP-FD gel 14 to 21 days after the initiation of the culture. These results strongly suggest that the XOL-transduced cells were successfully converted into dOBs in this 3D scaffold to form the fibronectin-coated NanoCliP-FD gel with dOBs (Fig. 2a).

Next we analyzed whether the dOBs produced calcified bone matrix in the fibronectin-coated NanoCliP-FD gel. As shown in Fig. 3b, Alizarin red S staining clearly showed massive formation of mineralized bone matrix in the fibronectin-coated NanoCliP-FD gel with dOBs, whereas non-transduced HDFs cultured under the same conditions only faintly did so. The results were confirmed by quantitative measurement of the staining intensity for the samples (Fig. 3c). The OsteoImage assay also showed a huge amount of calcium phosphate deposits in all over the fibronectin-coated NanoCliP-FD gel with dOBs (Fig. 3d), whereas the calcium phosphate was rarely deposited in the fibronectin-coated NanoCliP-FD gel with HDFs.

These results strongly suggest that calcified bone matrix was massively produced in the fibronectin-coated NanoCliP-FD gel with dOBs.

### Bone regeneration *in vivo* was promoted by the fibronectin-coated NanoCliP-FD gel with dOBs

**Figure 4:**
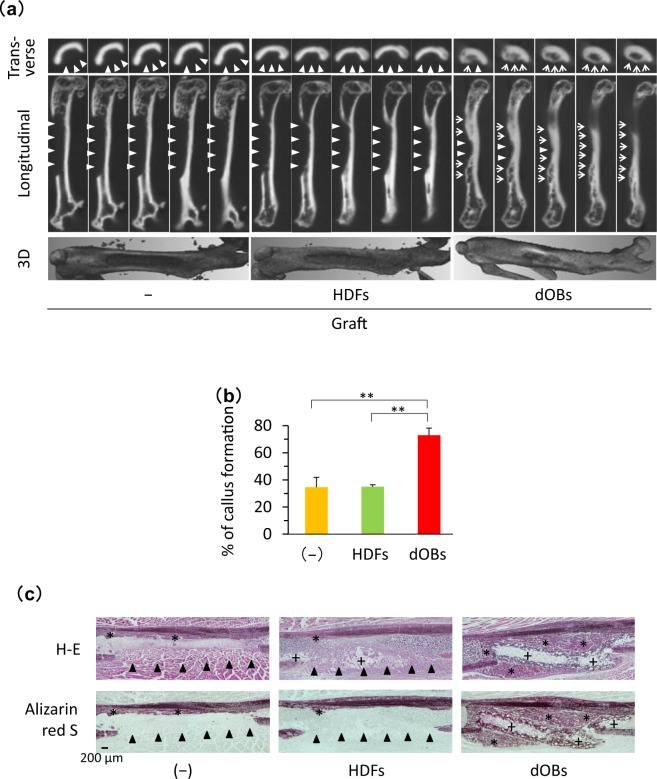
Bone healing was facilitated by transplantation of the fibronectin-coated NanoCliP-FD gel with dOBs. Fibronectin-coated NanoCliP-FD gel with HDFs or dOBs was prepared as in Fig. 3, and transplanted into an artificial segmental bone defect lesion that was created at femoral diaphysis in NOG/SCID mice. Control mice were not transplanted (−). Mice were sacrificed 21 days after the surgery. (**a**,**b**) µCT images of the femur were acquired. Serial 10-µm slices (top and middle) and 3D reconstructed (bottom) images (**a**) and %Callus formation (**b**) are shown. (**c**) Serial sections of the tissues were stained with H-E (upper) and Alizarin red S (lower). In (**a**) triangles and arrows represent bone defect lesions and regenerated bone tissue, respectively. In (**b**), values are means ± SD. N = 3 mice. **p < 0.01 vs. non-transplantation control. In (**c**), *and +represent regenerated bone tissue and NanoCliP-FD gel, respectively, and arrowheads represent bone defect lesions.

To estimate whether the fibronectin-coated NanoCliP-FD gel with dOBs can contribute to bone regeneration *in vivo*, we transplanted it into immune-deficient mice in which an artificial bone defect lesion was created at the femur site. Three weeks later, micro CT imaging revealed that the bone defect mostly remained in a control group of animals that were not transplanted as well as in another group that received transplantation of the fibronectin-coated NanoCliP-FD gel with HDFs (Fig. 4a). In contrast, transplantation of the fibronectin-coated NanoCliP-FD gel with dOBs resulted in the formation of a large callus that covered a large area of the femur leaving only small defect lesions. The different outcomes were statistically confirmed by calculation of percent of callus formation (Fig. 4b).

Histopathological examinations also confirmed that a large callus was formed to cover most of the area at the bone defect legion in mice transplanted with the NanoCliP-FD gel with dOBs (Fig. 4c). In contrast, a large defect remained at the femur transplanted with the NanoCliP-FD gel with HDFs.

These results strongly suggest that the NanoCliP-FD gel with dOBs promoted bone healing *in vivo* by producing regenerative bone tissue, while NanoCliP-FD gel with HDFs failed to do so.

### NanoCliP-FD gel did not cause any remarkable adverse event in mice

Fibronectin-coated NanoCliP-FD gel was transplanted into the subcutaneous tissue of mice, and 10 days later sera of the mice were subjected to laboratory tests. As shown in Supplementary Table S3, a significant difference was not detected between the mice and control mice with regard to all the items tested, strongly suggesting that the NanoCliP-FD gel was not toxic.

## Discussion

A variety of biomaterials have been developed so far to build 3D scaffolds for bone tissue engineering^20,21^. These include (i) bioceramics such as hydroxyl apatite^22^, (ii) natural macromolecules and their derivatives such as collagen, atelocollagen, chitosan and alginate^23–25^, (iii) synthetic polymers such as polyethylene glycol^26^, (iv) hydrogels^27,28^, and (v) other materials such as carbon nanotubes^29,30^. A variety of combinations of (i~v) have also been widely used^20,21,31–33^. They can be shaped into gel-like, sponge-like, mesh-like, or fibrous structures. In the present study, we constructed a novel nanogel tectonic material, the NanoCliP-FD gel, that is a kind of hydrogel fabricated by freeze-thawing and subsequent freeze-drying of chemically cross-linked nanogel consisting of natural macromolecules. The biocompatibility of NanoCliP gel in the use of implantation was evaluated in the previous study^8^. The H&E and immunohistochemical staining for monocytes and mature macrophages suggested that there was negligible histological evidence of a foreign body response after the implantation of NanoCliP gel. NanoCliP-FD matrix and gel consist of the same concentration of nanogels and cross-linkers and no xenogenic macromolecule is included in it, suggesting that the NanoCliP-FD gel is a biocompatible material. The NanoCliP-FD gel may be biocompatible and non-toxic. So far as we tested, a significant adverse event was not observed in mice transplanted with fibronectin-coated NanoCliP-FD gel (Supporting Information Table S3). A large amount of NanoCliP-FD gel can be easily and quickly prepared at a low cost.

In cell adhesion procedure, the solid-state NanoCliP-FD matrix was rehydrated by adding aqueous culture medium containing cells. The pore structures were still observed in NanoCliP-FD gel, which contains numerous large pores that were formed by fusion of pre-existing pores in the NanoCliP gel through freezing-and-drying (Fig. 1b). The enlarged pores allowed cells to easily penetrate into the gel and the cells forcibly contacted with the fibronectin-coated surface of the scaffold. From Fig. 2b and c, the cells effectively adhered onto the fibronectin-coated NanoCliP-FD gel, strongly suggesting that the fibronectin was effectively coated onto the surface of the walls of the expanded pores. The XOL-transduced cells proliferated and converted into dOBs in the NanoCliP-FD gel during the culture (Figs 2d and 3), which may be also due to the efficient adhesion of the cells onto the pores’ walls in the NanoCliP-FD gel. Therefore, this nanogel tectonic material exhibits excellent characteristics appropriate for a 3D scaffold for bone tissue engineering, and is efficiently combined with dOBs, thanks to the large pores formed by the freezing-and-drying treatment.

The NanoCliP-FD matrix is a dehydrated intermediate to produce NanoCliP-FD gel from the NanoCliP gel (Fig. 1a), and can be conveniently stored under a dry condition at room temperature for a long period. One may utilize the NanoCliP-FD matrix for storage and transport purposes, and the NanoCliP-FD gel can be quickly and easily prepared from the NanoCliP-FD matrix when necessary. This is a great advantage related to the utilization of the NanoCliP-FD gel for transplantation therapy, because NanoCliP-FD matrix can be stored and transported safely and at a low cost.

Compared with the fibronectin coating, the RGDC-conjugated NanoCliP-FD gel failed to provide the same efficient adherence of the dOBs onto the gel, so that the cells adhered to each other rather than to the pores’ walls and sphere-like aggregation of the cells appeared (Fig. 2c). The cells less efficiently adhered onto RGDC-conjugated NanoCliP-FD than onto fibronectin-conjugated one, probably because the RGCD was embeded/masked within the gel whereas the fibronectin was on the exterior. Fibronectin may not only allow adhesion of osteoblasts but also stimulate them. A recent report shows that the fibronectin isoforms containing the extradomain A may interact with α4β1 integrin to augment osteoblast differentiation. Other isoforms containing the extradomain B may interact with β3 integrin and promote osteoblast differentiation and mineralization^34^.

The NanoCliP-FD gel can be built to have any desired size and shape, because the cross-linking of CHPOA nanogel into NanoClik gel (Fig. 1a) can be processed in any template with the desired size and shape. The template can be fabricated in a computer-directed tailor-made fashion specific for the bone loss lesion in each patient.

In previous studies in which NanoClik gel was used as a scaffold for tissue engineering, the NanoClik gel was also used for slow release of some soluble factors, due to the molecular chaperon activity of the nanogel^35–37^. The soluble factors included BMP^35,36^, FGF18^36^, EP4A^35^, and W9 peptide^37^, which may have promoted bone regeneration *in vivo*. If such a slow release of soluble factors could also be achieved by means of the NanoCliP-FD gel, usefulness and efficacy of the gel in bone tissue regeneration may be further expanded due to the activities of the soluble factors. But this point need to be further examined in future studies.

Mesenchymal stem cells (MSCs) are widely used for bone tissue regeneration both *in vivo* and *ex vivo*, and remarkable therapeutic benefits of MSC transplantation have been confirmed in various clinical settings such as reconstruction of a large bone defect due to surgical resection of a bone tumor^38^. However, MSCs are harvested from bone marrow or adipose tissue of patients in a highly invasive manner. The number of MSCs harvested from a patient might not always be satisfactorily high, and proliferation and differentiation capacities of MSCs might be limited, especially in elderly cases^39,40^. In this respect, an additional option is required and the dOBs may be eligible as alternative cells to be used for transplantation therapy in bone diseases. A potential disadvantage of dOBs is that the dOBs are induced from fibroblasts by transducing Osterix, Oct3/4, and L-myc genes via retroviral vectors. The retroviral sequences integrated into the chromosomes may potentially cause aberrant expression of endogenous oncogenes to transform the dOBs into tumor cells. To overcome this problem, we developed a novel procedure to convert fibroblasts into osteoblasts by means of an addition of a chemical compound instead of gene transfer^41^. Such transgene-free dOBs may be more advantageous than the present ones, because the former may have further lower risk of tumor formation than the latter.

Taken together, the combination of the novel dOBs and the NanoCliP-FD gel may be a quite feasible approach in clinical applications for various bone diseases.

## Methods

### Preparation of NanoCliP-FD matrix

The NanoCliP gel was prepared as previously described (Fig. 1a)^8^. Briefly, the cholesterol-bearing pullulan (CHP), in which pullulan (average molecular weight = 1 × 10^5^ g/mol) was substituted with 1.2 cholesterol moieties per 100 anhydrous glucoside units, was purchased from NOF corporation (Tokyo, Japan). Acryloyl group-modified cholesterol-bearing pullulan (CHPOA) was synthesized using 2-Acryloyloxyethyl isocyanate (Showa Denko, Tokyo, Japan). CHPOA self-assembled into CHPOA nanogel, which was subsequently cross-linked by Michael addition of pentaerythritol tetra (mercaptoethyl) polyoxyethylene (PEGSH) (average molecular weight = 1 × 10^4^ g/mol) (NOF corporation) to form NanoClik gel in Microhematocrit Capillary Tubes with an inner diameter of 1.1 mm (Fisher Scientific) as a template. NanoClik gel was then converted into highly porous NanoCliP gel by freezing-induced phase separation. To prepare NanoCliP freeze-dried-matrix (NanoCliP-FD matrix), NanoCliP gel was quickly frozen in liquid nitrogen, followed by drying in a vacuum. Rhodamine-labeled NanoCliP gel and NanoCliP-FD matrix were prepared as above using acryloyl group-modified rhodamine-labeled CHP (CHPOA-Rh) instead of CHPOA, and rhodamine-labeled NanoCliP-FD matrix was soaked in PBS to form rhodamine-labeled NanoCliP-FD gel.

### Preparation of RGDC-conjugated and Fibronectin-coated NanoCliP-FD gel

RGDC conjugation was performed as follows. Synthetic RGDC (Arg-Gly-Asp-Cys) peptide (SCRUM Inc., Tokyo, Japan) was added to CHPOA nanogel simultaneously with the addition of PEGSH (Fig. 1a), in such a manner that final concentrations of CHPOA, PEGSH and RGDC peptide were 20 mg/mL, 35 mg/mL and 2 mg/mL, respectively. Subsequently, RGDC-conjugated NanoCliP gel and RGDC-conjugated NanoCliP-FD gel were prepared as above. For fibronectin-coating, NanoCliP-FD matrix was soaked in 50 μg/mL fibronectin solution (Wako laboratory chemicals, Osaka, Japan) for 6 hours, followed by rinsing twice in ethanol and drying (Fig. 1a). The resultant fibronectin-coated NanoCliP-FD matrix was hydrated to form fibronectin-coated NanoCliP-FD gel.

### Cells

Human dermal fibroblasts (HDFs) were purchased from DS Pharma Biomedical Co., Ltd. (Osaka, Japan) and cultured in Dulbecco’s minimum essential medium (DMEM) supplemented with 100 mM non-essential amino acids, 100 U/ml penicillin 100 μg/ml streptomycin, and 10% fetal bovine serum (FBS) in 5% CO_2_/95% humidified air at 37 °C (Standard culture conditions). Osterix (X), Oct3/4 (O) and L-myc (L) genes were transduced into the HDFs as described previously with slight modification^16^. Briefly, HDFs were seeded onto 100 mm culture dish at a density of 5 × 10^3^ cell/dish. Twenty-four hours later, cells were infected with a mixture of X, O and L retrovirus vectors freshly prepared using the PLAT-GP packaging cells (Toyobo, Osaka, Japan) as previously described^11,12^. After culturing for another 24 hours, the XOL-transduced cells were detached from culture dishes by trypsinization and resuspended in DMEM medium supplemented with 50 μg/mL ascorbic acid, 10 mM β-glycerol phosphate, 100 nM dexamethasone and 10% FBS (osteogenic medium).

### 3D culture

HDFs or XOL-transduced cells resuspended in the osteogenic medium were prepared in such a manner that the approximate cell density was 5 × 10^5^/50 μL. RGD-conjugated, Fibronectin-coated, and non-coated NanoCliP-FD matrices were soaked in the cell suspension, and air bubble was pushed out of the matrices with tweezers to allow infiltration of the cell suspension into the matrices (Fig. 1a). The matrices were incubated in the cell suspension for 2 hours. The resultant samples were transferred into 1.5 mL of fresh osteogenic medium in 12-well culture plates and cultured in 5% CO_2_/95% humidified air at 37 °C (the standard culture conditions).

### Calcein-AM staining

The samples were rinsed twice with PBS, and immersed in 0.1 mg/mL of calcein-AM (3′,6′-Di(O-acetyl)-2′,7′-bis[N,N-bis(carboxymethyl)aminomethyl]fluoresceinTetraacetoxymethyl Ester)(Dojindo, Kumamoto, Japan)/PBS solution for 20 min. The samples were rinsed twice with PBS, followed by fixation with 4% formaldehyde for 20 min. After three times washing with distilled water, the samples were observed under Confocal laser scanning microscope (CLSM) (LSM780; Carl Zeiss, Oberkochen, Germany)

### Phalloidin/Hoechst 33342 staining

The samples were rinsed with PBS, and fixed with 4% formaldehyde for 20 min. After washing with distilled water twice, the samples were immersed in 0.1% Triton X-100 for 5 min. The samples were further washed twice with distilled water, followed by staining with 0.1 mg/mL of Alexa Fluor® 488 phalloidin (Molecular Probes, Eugene, OR)/PBS solution for 20 min. After another three-times washing, the samples were observed under CLSM.

### Cell viability analysis

Cell viability was evaluated by a tetrazolium-based assay as previously described^42^. NanoCliP-FD gel with dOBs was soaked in 500 µL of osteogenic medium in 12-well plates and cultured under the standard culture conditions. One, 7 or 14 days later, the culture supernatant was replaced by 1,000 µl of fresh complete medium containing 2-(2-methoxy-4-nitrophenyl)-3-(4-nitrophenyl)-5-(2, 4-disulfophenyl)-2H-tetrazolium monosodium salt (WST-8; Nacalai Tesque, Kyoto, Japan) was added to the wells. After incubation for an additional 1 hour, the culture supernatant was harvested and OD at 490 nm was measured.

### Assessment of mineralization

For Alizarin red S staining, samples were fixed in 95% ethanol and stained with Alizarin red S solution (Sigma Aldrich) as previously described^16^. The OsteoImage mineralization assay was performed using the OsteoImage mineralization assay kit (Lonza) according to the manufacturer’s instruction. Briefly, the samples were rinsed with PBS, and fixed with 95% ethanol for 20 min. After washing, the samples were immersed in OsteoImage™ Staining Reagent for 30 min, followed by washing and observation under CLSM.

### Real time RT-PCR

Total RNA was extracted from cells using ISOGEN II (Nippon Gene Co. Ltd.) and reverse-transcribed using ReverTra Ace qPCR RT Master Mix (Toyobo). The resultant cDNA was mixed with Real-Time PCR Master Mix (Applied Biosystems, Waltham, MA) and matching probes/primers specific for human β-actin, osteocalcin and osteopontin genes (Supporting Information Table S2)). Real time PCR was carried out on a 7300 Real Time PCR System (Applied Biosystems). Values (means ± SD) were normalized with respect to the β-actin mRNA level in each sample, and relative values were calculated.

### Surgery and transplantation

All experimental procedures and protocols for animals conformed to the National Institutes of Health Guide for the Care and Use of Laboratory Animals and were approved by either the Committee for Animal Research of Kyoto Prefectural University of Medicine or the Animal Care. dOBs were induced from HDFs, seeded into fibronectin-coated NanoCliP-FD gel (1 × 1 × 6 mm), and cultured for 7 days as above. After culturing for 7 days, some sample was tested for bone matrix formation by Alizarin red S staining, and the other samples were used for transplantation. Six-week-old male NOG/SCID mice were anesthetized with isoflurane and a segmental bone defect with a size of 1 × 1 × 6 mm was created at the diaphysis of the left femur using a dental drill under pouring water.

### Radiological and histological analysis

Twenty-one days after the transplantation, mice were euthanized with a lethal dose of isoflurane. Thighs were dissected, fixed with 10% neutral-buffered formalin, and subjected to μCT imaging (Toshiba). Specimens were embedded in SCEM compound (Leica Microsystems), quick-frozen, and cryo-sectioned into 6-μm slices. After staining with H & E and Alizarin Red S, the sections were analyzed with BZ-X710 fluorescence microscope (Keyence).

### Statistical analysis

Data are expressed as means ± standard deviation (S.D.). Statistical significance was analyzed using Student’s t-test. P < 0.05 was considered significant.

## Electronic supplementary material

Supplementary Tables S1–3
