# Supplementary material for: Nanogel tectonic porous 3D scaffold for direct reprogramming fibroblasts into osteoblasts and bone regeneration
Source: Sci Rep. 2018 Oct 25;8:15824. doi: 10.1038/s41598-018-33892-z (PMC6202359; doi:10.1038/s41598-018-33892-z)
Supplement: Supplementary file 1 — Supplementary Tables S1–3 [file 41598_2018_33892_MOESM1_ESM.docx]

**Supporting Information**

Nanogel tectonic porous 3D scaffold for direct reprogramming fibroblasts into osteoblasts and bone regeneration

Yoshiki Sato ^a,b^, Kenta Yamamoto^a,b^, Satoshi Horiguchi ^a,b^, Tahara Yoshiro ^c^, Kei Nakai ^a,b^, Shin-ichiro Kotani ^a^, Fumishige Oseko ^b^, Giuseppe Pezzotti ^a,d^, Toshiro Yamamoto ^b^, Tsunao Kishida ^a^, Narisato Kanamura ^b^, Kazunari Akiyoshi ^c*^, Osam Mazda ^a*^

^a^ Department of Immunology, Kyoto Prefectural University of Medicine, Kyoto Japan

^b^ Department of Dental Medicine, Kyoto Prefectural University of Medicine, Kyoto Japan

^c^ Department of Polymer Chemistry, Graduate school of engineering, Kyoto University, Kyoto Japan

^d^ Ceramic Physics Laboratory, Kyoto Institute of Technology, Kyoto Japan

|  | NanoClik gel | NanoCliP gel | NanoCliP FD-gel |
| --- | --- | --- | --- |
| Water content (wt%) | 97.1 ± 0.1 | 97.9 ± 2.0 | 95.0 ± 0.1 |
| Porosity | N.D. | 54.2 ± 4.9 | 81.9 ± 1.1 |

**Supporting Information Table S1**

Characterization of Cross-linked nanogel. Porosity was calculated based on the CLSM images observed by two-photon laser at 820 nm. N.D., not determined.

| Target | Supplier | Catalog number |
| --- | --- | --- |
| Human β-actin | Applied Bioscience | Hs01060665_g1 |
| Human osteocalcin | Applied Bioscience | Hs01587814_g1 |
| Human osteopontin | Applied Bioscience | Hs00959010_m1 |

**Supporting Information Table S2**

Primers used for real-time RT-PCR in this study.

|  | Transplantation | | P value  (t-test) |
| --- | --- | --- | --- |
|  | (-) | (+) |  |
| TP (g/dL) | 5.0 ± 0.0 | 5.0 ± 0.0 | N.A. |
| ALB (g/dL) | 3.8 ± 0.1 | 3.8 ± 0.1 | >0.05 |
| BUN (mg/dL) | 27.4 ± 0.3 | 27.0 ± 0.2 | >0.05 |
| CRE (mg/dL) | 0.2 ± 0.0 | 0.2 ± 0.0 | >0.05 |
| Na (mEq/L) | 155.0 ± 1.15 | 154.0 ± 0.0 | >0.05 |
| K (mEq/L) | 6.7 ± 0.1 | 6.6 ± 0.0 | >0.05 |
| Cl (mEq/L) | 106.5 ± 1.0 | 105.5 ± 1.0 | >0.05 |
| Ca (mg/dL) | 9.1 ± 0.1 | 9.0 ± 0.0 | >0.05 |
| IP (mg/dL) | 8.1 ± 0.1 | 8.2 ± 0.1 | >0.05 |
| AST (IU/L) | 193.5 ± 4.7 | 191.0 ± 1.2 | >0.05 |
| ALT (IU/L) | 33.0 ± 2.0 | 33.5 ± 1.0 | >0.05 |
| LDH (IU/L) | 1,086 ± 7.7 | 1,075 ± 3.8 | >0.05 |
| AMY (IU/L) | 2,157 ± 25.0 | 2,146 ± 8.2 | >0.05 |
| T-CHO (mg/dL) | 77.5 ± 1.0 | 77.5 ± 3.0 | >0.05 |
| TG (mg/dL) | 62.5 ± 3.8 | 60.5 ± 1.9 | >0.05 |
| HDL-C (mg/dL) | 43.0 ± 1.2 | 42.5 ± 1.0 | >0.05 |
| T-BIL (mg/dL) | 0.10 ± 0.01 | 0.10 ± 0.01 | >0.05 |
| GLU (mg/dL) | 155.5 ± 1.9 | 153.0 ± 1.2 | >0.05 |

**Supporting Information Table S3**

Eight-week-old female C57BL/6 mice were subcutaneously transplanted with fibronectin-coated NanoCliP FD-gel (1 x 1 x 10 mm in size) into the flank (+), while control mice were sham operated (-). Ten days later, sera were collected, and the indicated results were obtained. N.A., not applicable. N=4 mice for each group
